# Angiotensin Type 2 Receptor Gene Polymorphisms and Susceptibility to Preeclampsia

**Published:** 2018

**Authors:** Mohammad Sadegh Soltani-Zangbar, Behnoush Pahlavani, Jaleh Zolghadri, Behrouz Gharesi-Fard

**Affiliations:** 1- Department of Immunology, School of Medicine, Shiraz University of Medical Sciences, Shiraz, Iran; 2- Islamic Azad University, Fars Science and Research Branch, Shiraz, Iran; 3- Infertility Research Center, Shiraz University of Medical Sciences, Shiraz, Iran; 4- Department of Obstetrics and Gynecology, School of Medicine, Shiraz University of Medical Sciences, Shiraz, Iran

**Keywords:** AT1R, AT2R, Polymorphism, Preeclampsia

## Abstract

**Background:**

The purpose of the study was to determine the relationship between angiotensin II type 1 receptor at position+1166 (AT1R+1166A/C; rs5186) and angiotensin II type 2 receptor at position+1675 (AT2R+1675A/G; rs5194) gene polymorphisms with preeclampsia in an Iranian women population.

**Methods:**

430 women were recruited in this study including 212 preeclamptics and 218 healthy women. PCR-RFLP method was used for genotyping the polymorphisms. Chi square and Fisher exact test were used for comparing case and control groups. The p<0.05 was considered statistically significant.

**Results:**

The frequency of genotypes of the AT1R gene and AT2R gene was similar in preeclampsia and normal pregnancy. There were no significant differences in genotype and also allele frequencies between preeclamptics and healthy women regarding the two studied polymorphisms. AT1R/AT2R genotypes combination study indicated that there was a statistically significant difference between preeclamptics and healthy women. AC/AG combination was significantly decreased, while CC/AA combination showed significant increase in patients compared with the healthy women (p<0.01).

**Conclusion:**

The present study showed that the genetic polymorphisms within AT1R and AT2R genes may be associated with susceptibility to preeclampsia in Iranian women.

## Introduction

Preeclampsia (PE) is known as hypertension during pregnancy along with the presence of proteinuria after the 20th week of gestation (1). Preeclampsia is a multisystem and one of the most common and serious pregnancy diseases that affects approximately 5% of all human pregnancies and in up to 10% to 20% of nulliparous women (2–4). Although the exact etiology of this disease has still remained unknown but several causes have been reported that may have a possible role in the development of PE, including abnormal placental implantation, feto-placental intolerance, endothelial dysfunction and genetic factors (5, 6). When convulsion or coma is added to hypertension and proteinuria, the disease is named eclampsia (7). Hypertension is the main sign of PE and the renin-angiotensin system (RAS) has an important role in the regulation of blood pressure during pregnancy and pathophysiology of PE (8, 9). The active component of RAS is the peptide hormone angiotensin II (10). In this system, angiotensin II via two distinct receptors, angiotensin II type-1 (AT1R) and angiotensin II type-2 (AT2R) regulates the blood pressure (6, 8). It is believed that the majority of angiotensin II functions are mediated by AT1R, including vasoconstriction and cellular growth and proliferation (10). AT1 receptor is expressed by vascular smooth muscle and adrenal gland cells while the AT2 receptor is reported to be present in the fetal tissues, uterus, brain and adrenal medulla (6, 11). Interaction of angiotensin II with AT2R induces vasodilation and inhibits growth and cell proliferation and differentiation (12). Both receptors contain single nucleotide gene polymorphism which are reported to be associated with receptors function (13). AT1R gene is located on chromosome 3 and a single nucleotide polymorphism (+1166A/C; rs5186) has been shown within 3′ untranslated region of the gene (10). AT2R gene is located on the X chromosome and a single nucleotide gene polymorphism (+1675G/A; rs5194) within coding region at position +1675 has been defined that is related to gene transcription and translation start point (14, 15).

Previous studies showed that different polymorphisms in AT1R and AT2R gens have various effects on hypertension (15, 16). Statistical association between +1166A/C polymorphisms and hypertension has been reported by several studies (8, 9). There are some findings that AT1R (+1166A/C) and AT2R (+1675A/G) polymorphisms have significant association with PE in some ethnic groups such as Afro-Caribbean women but not in others (2, 8, 10, 17). The present study aimed at investigating the association between AT1R (+1166A/C) and AT2R (+1675G/A) gene polymorphisms and susceptibility to PE in a group of Iranian women due to controversial results from different gene polymorphism studies in various ethnic groups.

## Methods

### Subjects:

A total of 430 women were recruited in this study including 212 preeclamptics and 218 healthy women. Inclusion criteria for PE patients were systolic blood pressure of ≥140 *mmHg* or diastolic blood pressure of ≥90 *mmHg* along with the presence of more than 0.3 *gr* proteins in a 24 *hr* urine collection or at least 1+ protein or greater urine dipstick after 20 weeks of gestation. Healthy women were selected among volunteer pregnant women without any history of autoimmunity, malignancy, hypertension or family history for PE. All participants were selected among pregnant women referred to Hafez and Zeynabiyeh hospital (affiliated to Shiraz University of Medical Sciences, Shiraz-Iran) or Emam-Hasan Mojtaba hospital (Darab-Iran). Informed consent was taken from all participants and the method of this study was approved by the local Ethics Committee of Shiraz University of Medical Sciences, Shiraz, Iran.

### Genotyping:

Genomic DNA was extracted from blood samples using a DNA isolation kit (Genet Bio, Korea). Polymerase Chain Reaction –Restriction Fragment Length Polymorphism (PCR-RFLP) method was used for genotyping. For genotyping of AT1R +1166A/C, the following primers were used to amplify a 350 *bp* DNA product: forward 5′-ATAATGTAAGCTCATCCACC-3′ and reverse 5′-GAGATTGCATTTCTGTCAGT-3′ (Generay Biotech, China, (8)). The quality of PCR products were assessed by agarose gel electrophoresis and then subjected to fragmentation with one unit of DdeI as restriction enzyme (Fermentas, Lithuania) to create two bands of 210 and 140 *bp* based on the genotype. A 310 *bp* PCR product was amplified for AT2R gene (+1675A/G) using forward 5′-AGAGATCTGGTGCTATTACG-3′ and reverse 5′-CACTTGAAGACTTACTGGTTG-3′ primers (Generay Biotech, China, (8)) and digested with HPY 188 III (New Englan Biolabs, UK). After enzymatic digestion, two bands of 206 and 104 *bps* were created. The details of PCR-RFLP conditions are indicated in table 1.

**Table 1.** PCR-RFLP conditions for AT1R (+1166A/C) and AT2R (+1675A/G)

**Locus**	**PCR condition**	**Reference**
**AT1R (+1166A/C)**	35 cycle: 94°*C* 45 *s*, 55°*C* 60 *s*, 72°*C* 60 *s* The products were digested by 5 *U* DdeI at 37°*C* for 2 *hr*	8
**AT2R (+1675A/G)**	35 cycle: 94°*C* 45 *s*, 55°*C* 60 *s*, 72°*C* 60 *s* The products were digested by 5 *U* HYP 188 III at 37°*C* >3 *hr*	8

### Statistical analysis:

Statistical evaluation was carried out using the statistical package for SPSS 16 for windows (SPSS Inc., Chicago, IL, USA). The frequencies of alleles/genotypes were compared in cases and controls by chi-square test and Fisher exact test when appropriate. All reported p-values were two-tailed with a confidence interval (CI) of 95%.

## Results

The clinical characteristics of PE cases and healthy controls are presented in table 2. Statistical analysis indicated that there was no significant statistical difference between two groups regarding maternal and gestational age while as expected systolic and diastolic blood pressure was significantly higher in PE cases compared to healthy controls.

**Table 2.** Demographic characteristics of PE patients and healthy controls (Mean±SD)

**Groups**	**Age**	**Gestational age**	**SBP (*mm Hg*)**	**DBP (*mm Hg*)**
**Preeclampsia (n=212)**	28.3±4.1	32.1±3.1	148.9±15.3^*^	97.3±9.8^*^
**Control (n=218)**	27.3±3.7	33.4±3.8	108±11.5	78±7.8

* p<0.05

The distribution of genotype and allele frequencies for the AT1R and AT2R genes were compared between preeclamptic patients and healthy women (Table 3). While PE cases for both SNPs were in hardy-Weinberg equilibrium, control groups for both studied SNPs were not in hardy-Weinberg equilibrium. The sample size might account as the main reason for this disequilibrium.

**Table 3.** Allele and genotype frequencies of AT1R (+1166A/C) and AT2R (+1675G/A) gene polymorphisms in preeclampsia patients and controls

**ATR SNPs**	**Allele**	**Control, n (%) (n=436)**	**Patients, n (%) (n=424)**	**p-value**	**Genotype**	**Control, n (%) (n=218)**	**Patients, n (%) (n=212)**	**p-value**
**AT1R (+1166A/C)**	A_1_ C	301(69.04%) 135(30.96%)	283(66.75%) 141(33.25%)	0.52	AA CC AC	143(65.60%) 60(27.52%) 15(6.88%)	136(64.15%) 65(30.66%) 11(5.19%)	0.64^*^

**AT2R (+1675A/G)**	A_2_ G	232(53.21%) 204(46.79%)	235(55.42%) 189(44.58%)	0.56	AA GG AG	63(28.90%) 49(24.48%) 106(48.62%)	75(35.38%) 52(24.53%) 85(40.09%)	0.19^*^

SNP: Single nucleotide polymorphism, p-value calculated using chi-square test; 1: In +1166A/C site common allele and genotype are A and AA; 2: In +1675A/G site common allele and genotype are A and AA;

* The power of study for both SNPs were over 73%

As indicated in table 3, the frequencies of AA, AC and CC genotype of +1166A/C polymorphism of the AT1R gene in patients and healthy pregnant women have no significant difference (Table 3, p=0.64). Moreover, the A and C allele’s frequencies of this polymorphism were also compared between patients and healthy women. Regarding the genotypes, there were no statistical differences in alleles distribution between preeclamptic patients and healthy pregnant women (Table 3, p=0.52).

Regarding +1675G/A polymorphism shown in table 3, the frequencies of AA, AG, and GG genotypes in patients and controls had no significant differences in genotypes and alleles frequencies between patients and controls (p=0.19, and p= 0.56, respectively).

Statistically significant differences have been observed in combination study of the AT1R/AT2R genotypes. AC/AG combination significantly decreased, while CC/AA combination showed significant increasing in patients as compared with the healthy women (p<0.01, Table 4).

**Table 4.** Combined genotype of AT1R/AT2R genes in preeclampsia compared with healthy women

**AT1R/AT2R genotypes**	**Control (n=218)**	**Preeclampsia (n=212)**	**p-value**
**AA/AA**	47	45	0.01
**AA/GG**	34	31	
**AA/AG**	62	60	
**AC/AA**	0	5	
**AC/GG**	0	3	
**AC/AG**	15	3	
**CC/AA**	16	25	
**CC/GG**	15	18	
**CC/AG**	29	22	

## Discussion

Several genetic, environmental, immunological and physiological factors have been shown to play role in susceptibility to preeclampsia (1, 18–20). Since hypertension is one of the important symptoms in preeclamptic women, reasons underling this fact may reveal the etiology of this disease. Regulative function of renin-angiotensin system in blood vessel diameter probably plays an essential role in blood pressure regulation and so in preeclampsia (8, 9). RAS regulates the blood pressure by two receptors of angiotensin II receptor 1 (AT1R) and angiotensin II receptor 2 (AT2R) (6, 8). Expression of AT1R and AT2R has been reported in various cells and tissues like smooth muscles, adrenal gland cells, brain, uterus and fetal tissues (6, 8). Different genetic variations may impress the expression of these receptors. AT1R and AT2R genes which are located on distinct chromosomes and various single nucleotide polymorphisms have been reported in coding and noncoding parts of these genes’ sequences. Approximately, 50 and more than 4 SNPs for AT1R and AT2R genes have been reported, respectively (10, 14, 15, 21).

In this study, the gene polymorphisms of AT1R (+1166A/C; rs5186) and AT2R (+1675A/G; rs5194) with preeclampsia were investigated in healthy pregnant and preeclamptic women. 212 preeclamptic and 218 healthy pregnant women took part as case and control population. Our results indicated that there was no significant association between AT1R (+1166A/C) gene polymorphism in PE and healthy pregnant women in our Iranian group of study. These findings have been reported by other studies in different populations. Akbar et al. in their study outlined that there was no association between AT1R +1166A/C gene polymorphism and PE in Afro-Caribbean, Caucasian and Asian women (8). Also, Alkanli et al. didn't find any association among PE and AT1R A/C polymorphism in Turkish females (22). Salimi et al. in a study on Afghan, Balooch and Persian females didn't find any relation among AT1R A/C gene polymorphism and PE (6). The results of AT1R polymorphisms and PE are controversial; in the study of Bouba et al., significant association between +1166C variant and PE was reported (23). Also, associations of AT1R and hypertension in pregnancy were reported in Japanese and Polish populations (24, 25). Because of different reported results, ethnic variations and the environmental difference may have an intervening role in this controversy.

Regarding AT2R (+1675A/G) gene polymorphism, the association between PE and AT2R gene polymorphisms also remains controversial. A significant association between GG genotype of AT2R gene in +1675 position and PE has been reported in Afro-Caribbean and Caucasian women by Akbar et al. which was the first report of such an association for Afro-Caribbean population (8). Zhua et al. reported the same results in Chinese women (26). The association between PE and other SNPs of AT2R such as C4599A had been shown in other studies (10, 15). Probably, similar to AT1R gene polymorphisms, the association of AT2R SNPs and PE is also influenced by ethnic and environmental factors.

A significant association between combined genotypes of AT1R AC/AT2R AG and PE has been outlined in our study. In line with this finding, Akbar et al. have reported an association of combined AT1RAC/AT2RAG variants in preeclamptic Afro-Caribbean toward healthy pregnant women (8). The combination of these SNPs in several genes may synergically raise the preeclampsia risk (6). Our results are consistent with mentioned studies.

Since hypertension is one of the main symptoms of preeclampsia and the rennin-angiotensin system has an important role in blood pressure regulation, genetic factors such as various SNPs in different RAS genes may affect its expression leading to blood pressure and susceptibility to preeclampsia. Regarding these reasons, numerous studies about RAS gene's polymorphisms especially, AT1R, AT2R, and PE have been performed, and confusingly, the results are controversial.

Small sample size and lack of studies regarding the functional mechanisms of AT1R, AT2R polymorphisms are two main limitations of this study.

## Conclusion

The results of the present study indicated that none of the studied polymorphisms alone are associated with PE but the combination of two polymorphisms might be associated with susceptibility to PE in Iranian women. It seems that the combination of genetic polymorphisms within different important genes, including AT1R and AT2R, might account as genetic factors for susceptibility to PE. A comprehensive study of combination polymorphisms within various genes may provide more information about the genetic causes of preeclampsia in the future.
